# Acute Dilation of Venous Sinuses in Animal Models of Mild Traumatic Brain Injury Detected Using 9.4T MRI

**DOI:** 10.3389/fneur.2020.00307

**Published:** 2020-04-28

**Authors:** Qandeel Shafqat, Jennaya Christensen, A. Max Hamilton, Elizabeth Imhof, Richelle M. Mychasiuk, Jeff F. Dunn

**Affiliations:** ^1^Department of Radiology, Faculty of Medicine, University of Calgary, Calgary, AB, Canada; ^2^Hotchkiss Brain Institute, Faculty of Medicine, University of Calgary, Calgary, AB, Canada; ^3^Department of Clinical Neurosciences, Faculty of Medicine, University of Calgary, Calgary, AB, Canada; ^4^Alberta Children's Hospital Research Institute, Faculty of Medicine, University of Calgary, Calgary, AB, Canada; ^5^Department of Psychology, Faculty of Arts, University of Calgary, Calgary, AB, Canada; ^6^Department of Neuroscience, Central Clinical School, Monash University, Melbourne, VIC, Australia

**Keywords:** transverse sinuses (TS), sinus dilation, venous drainage, superior sagittal sinus (SSS), MRI-magnetic resonance imaging, mild traumatic brain injury (mTBI), concussion, animal model

## Abstract

Mild traumatic brain injury (mTBI) is a debilitating but extremely common form of brain injury that affects a substantial number of people each year. mTBI is especially common in children and adolescents. Our understanding of mTBI pathophysiology is limited, and there is currently no accepted marker for disease severity. A potential marker for disease severity may be cerebrovascular dysfunction. Recent findings have implicated cerebrovascular alteration as an important component of mTBI and suggest it contributes to the development of persistent, long-term symptoms. In this paper, we conducted two studies to investigate whether mTBI affects venous drainage patterns in the central nervous system using alterations in the size of venous sinuses as a marker of changes in drainage. Using a closed head vertical weight-drop model and a lateral impact injury model of mTBI, we imaged and quantified the size of three major draining vessels in the adolescent rat brain using 9.4T MRI. Areas and volumes were quantified in the superior sagittal sinus and left and right transverse sinuses using images acquired from T2w MRI in one study and post-gadolinium T1w MRI in another. Our results indicated that the three venous sinuses were significantly larger in mTBI rats as compared to sham rats 1-day post injury but recovered to normal size 2 weeks after. Acutely enlarged sinuses post-mTBI may indicate abnormal venous drainage, and this could be suggestive of a cerebrovascular response to trauma.

## Introduction

Mild traumatic brain injury (mTBI) is one of the most common forms of acute brain injury, affecting an estimated 42 million people each year (1, 2). This is especially a problem for children and adolescents (3, 4), as mTBIs that occur during critical periods of brain development may lead to long-term neurological symptoms (5). There is a wide range of symptoms associated with mTBI which can last for hours to years (6, 7). Currently, there is no accepted method of detecting mTBI with imaging. Both structural and vascular related studies have shown promise (8–10). It is important to fully understand the pathophysiology of mTBI due to the complexity of mTBI, the need for biomarkers, and the quest for treatment options.

Most MRI cerebrovascular studies to date relate to perfusion or susceptibility. There is evidence for perfusion changes in mTBI (11–14). Cerebral blood flow (CBF) studies have reported varied results with both increases and decreases in CBF being measured at multiple points post injury (14, 15). Some studies found an association between CBF and persistent mTBI symptoms (10, 15). This association could explain the heterogeneity in CBF findings as patient symptoms vary between studies. Given the mixed results, further studies are needed to understand the role of cerebrovascular alterations in mTBI pathophysiology.

One understudied area of cerebrovascular alteration in mTBI is venous regulation. A decrease in magnetic susceptibility of the straight sinus has been observed using 3.0T MRI in mTBI patients (12). This decrease in susceptibility was suggestive of an increase in cerebral venous oxygen saturation and correlated with symptom severity (12). Increased oxygen saturation may be due to increases in CBF, or reductions in oxygen metabolism in the brain following injury. Decreased flow in the internal jugular veins, measured with MR venography has been observed in mTBI patients (16). These changes occurred alongside an increase in intracranial pressure and a decrease in intracranial compliance in mTBI.

We propose that these studies provide sufficient evidence to indicate that there could be changes in venous flow regulation post-mTBI. The purpose of this paper was to study venous drainage following adolescent mTBI. Changes in volume and cross-sectional area of the superior sagittal sinus (SSS) and the left and right transverse sinuses (LTS, RTS) were a metric of alterations in venous drainage, as these sinuses regulate a substantial amount of cerebral venous drainage. Two rat models of mTBI, a vertical weight-drop model and a lateral impact model, were utilized to reduce the potential of the injury location being a confounding factor. We hypothesized that post-mTBI rats will experience cerebrovascular alterations which would impact venous drainage, thereby altering the sinus size.

## Methods

### Animals

Sprague-Dawley rats (70–130 g) bred in house were maintained on a 12-h light/dark cycle with *ad-libitum* access to food and water. Pups were weaned at P21 and experiments began at P30. Experiments were conducted in accordance to principles outlined in the current Guidelines of the Canadian Council of Animal Care. The Animal Care Committee at the University of Calgary approved ethics for Study 1, and the University of Calgary Conjoint Faculties Research Ethics board for Study 2.

### mTBI Administration

Study 1: mTBI was induced in rats (*n* = 12; 5 males, 7 females) at P30 using a vertical weight-drop model technique as described (17). In brief, all rats were anesthetized using 3–4% isoflurane until unresponsive to a toe pinch and placed on a sheet of aluminum foil suspended 10 cm above a sponge cushion. mTBI was induced by dropping a brass weight from a height of 0.5 m through a plexiglass tube. The aluminum foil broke upon impact, causing the rat head to rotate. Topical lidocaine was applied on the injury site. Sham rats (*n* = 5; 3 males, 2 females) received the same treatment, including anesthetic, without the impact.

Study 2: Four female pups (P21) were housed per cage. Rats from each cage were randomly assigned to the repeated mTBI group (*n* = 14) or the sham-injury group (*n* = 13). A lateral impact model was used for mTBI induction as described (18). Briefly, rats received three mTBIs, each single mTBI spaced 3 days apart. After anesthetization with 2–5% isoflurane, rats were placed in a prone position with the left side of the head resting against a protective headguard. A 50 g weight was propelled at an average speed of 9.02 m/s ± 0.18 toward the left temporal lobe to induce injury. Rats received topical lidocaine at the injury site and were transferred to a heated cage for recovery. Sham rats received the same treatment under anesthetic, without the impact.

### Behavioral Testing

Injury was validated using time-to-right (TTR) (17). TTR is a measure of the amount of time taken by the rats to move from a supine to a prone position immediately after the impact. Since we utilized repeated impact in Study 2, TTR was measured after each injury to obtain three measurements. The three measurements were averaged to get one TTR value per rat.

### MRI Image Acquisition

MR data was acquired using a 9.4T MRI Bruker Avance-console and a 35 mm quadrature volume coil.

Study 1: Rats were anesthetized with isoflurane and imaged 24–30 h post-mTBI. Images were acquired using a rapid acquisition with relaxation enhancement (RARE) sequence (TE_eff_ = 32 ms, TR = 4,000 ms, RARE factor: 8, FOV = 25.6 × 25.6 mm, matrix: 256 × 256, voxel resolution: 0.1 × 0.1 × 1mm, number of slices: 20).

Study 2: The sham and mTBI rats from Study 2 were divided into two imaging groups. Rats from the first group were imaged 1 day after the third mTBI (sham *n* = 6; mTBI *n* = 7). The second group was imaged 2 weeks after the third mTBI (sham *n* = 7; mTBI *n* = 7).

Prior to imaging, rats were anesthetized with 100 mg/kg of ketamine/xylazine, placed into a stereotaxic frame and given an injection of 80 ul of 0.03 mM gadolinium into the Cisterna magna. Images were taken 2.5 h post-injection initiation. A gradient echo flow compensated (GEFC) sequence was used to acquire images (TE = 2.6 ms, TR = 18 ms; Averages = 1, flip angle = 15, scan time = 4m12s, FOV=32 × 28 × 20 mm, matrix=320 × 140 × 100, voxel resolution=0.2 × 0.2 × 0.2 mm, number of slices: 100).

### MRI Data Processing

Study 1: T2-weighted RARE anatomical images were used. The superior sagittal sinus (SSS), and the left and right transverse sinuses (LTS, RTS) were measured. SSS volume was measured using 3D Slicer (version 4.6.0). Bruker data were first converted to DICOM. An anatomical marker for segmentation was defined to ensure a consistent start and end point for all rats. A blood vessel was observed below the SSS which separated as the slices moved toward the anterior part of the brain. The segmentation began at the slice in which this blood vessel clearly separated from the sinus and ended after 10 mm of slices (10 consecutive slices). Segmentations were used to create 3D models for volumes and visualization in 3D Slicer.

The image resolution was not high enough to segment the LTS and RTS using 3D Slicer, so we also included cross-sectional area measurements for all three sinuses. ImageJ software was used for area measurements. Regions of interest (ROIs) were drawn around the SSS, LTS, and RTS on 3 consecutive slices at approximately bregma −2.40, −3.40, and −4.40 based on a postnatal (P21) rat brain atlas (19). The areas measured from the ROIs of the 3 slices were averaged to obtain one measurement per sinus.

Study 2: T1-weighted GEFC images were used to analyze the SSS. The LTS and RTS were not analyzed due to lower resolution. We had a more comprehensive set of MR data (100 slices) in this study, and so chose to measure SSS volume using 3D Slicer. 3D models for the SSS were developed using the same methods and anatomical marker from Study 1. The only difference was that we segmented the sinus on 50 consecutive slices in order to cover the 10 mm of slices.

### Statistical Analyses

SPSS 25.0 (SPSS Inc. Chicago, Ill., USA) software was used for statistical analyses. The significance threshold was set at *p* < 0.05.

Study 1: Areas and volumes were compared between injury groups using Student's *t*-tests.

Study 2: Rats were divided into four groups based on injury and imaging timepoint (sham 1-day *n* = 6; sham 2-wks *n* = 7; mTBI 1-day *n* = 7; mTBI 2-wks *n* = 7). Volumes were compared using a two-way ANOVA with a Tukey *post-hoc* test.

## Results

### Behavioral Results From Both Studies Confirm Successful mTBI

**Figure 1:**
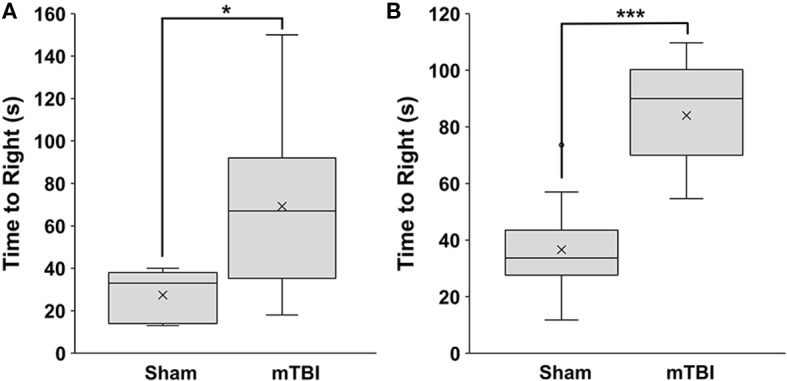
Time to right measured immediately post-mTBI or sham treatment. **(A)** Study 1 (mean±SD; sham *n* = 5; mTBI *n* = 12) and **(B)** Study 2 (mean±SD; sham *n* = 13; mTBI *n* = 14). Comparisons were done with a students *t*-test, *p < 0.05, ***p < 0.001.

mTBI rats had a significantly longer time-to-right than sham rats, at 69 ± 39 s and 27 ± 13 s, respectively (*p* = 0.037) in Study 1 (Figure 1). Similar results were seen in Study 2, with the time-to-right for mTBI rats being 84 ± 18 s, and sham rats being 37 ± 16 s (*p* = 9.5 × 10^−8^). There was no significant difference (*p* = 0.22) between time-to-rights for the sham groups from Study 1 and Study 2 (Figure 1).

### mTBI Rats From Study 1 Have Enlarged Sinuses 1-Day Post-injury

**Figure 2:**
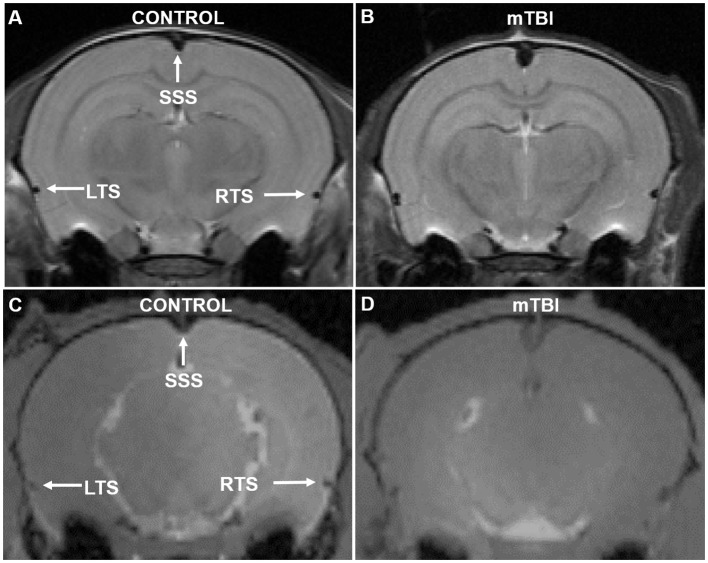
Example MR images from Study 1 **(A,B)** and Study 2 **(C,D)** of a control and mTBI rat 1-day post injury. **(A,B)** High-resolution T2w RARE images (TR/TE= 4,000/16, RARE factor=8, voxel resolution= 0.1 × 0.1 × 1 mm). **(C,D)** T1w GEFC images (TE = 2.6 ms, TR = 18 ms, voxel resolution = 0.2 × 0.2 × 0.2 mm). Enlargement of the superior sagittal sinus (SSS) is readily apparent. The LTS and RTS appear larger in mTBI vs. sham rats in Study 1 **(A,B)**. The LTS and RTS were not analyzed in Study 2 due to lower resolution.

Enlarged sinuses, especially the superior sagittal sinus, are seen on anatomical MRIs of mTBI rats (Figure 2).

**Figure 3:**
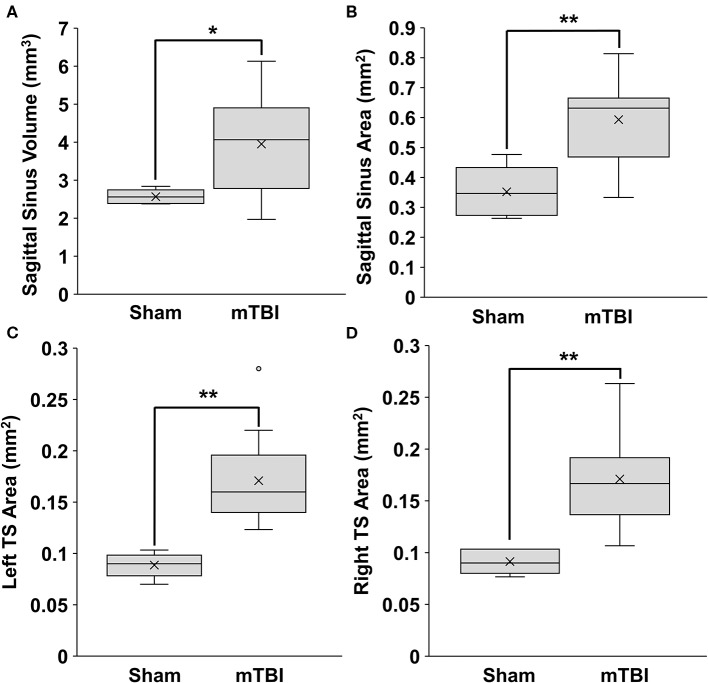
Sinus volumes and areas of rats 1-day post injury from Study 1. Data were obtained from T2w MRI. Volumes were calculated from 10 mm of consecutive slices. Cross-sectional areas were calculated as an average from 3 consecutive slices. **(A)** Superior sagittal sinus (SSS) volume, **(B)** SSS area. **(C)** Left transverse sinus (TS) area. **(D)** Right TS area. (mean±SD; sham *n* = 5; mTBI *n* = 12). Comparisons were done with a students *t*-test, *p < 0.05, **p < 0.01.

The SSS volume was significantly increased in mTBI rats as compared to sham rats, with values at 4.0 ± 1.2 and 2.6 ± 0.19 mm^3^, respectively (*p* = 0.024) (Figure 3). The area measurement for the SSS showed similar results, with mTBI rats having a larger cross-sectional area than the sham rats (0.59 ± 0.14 mm^2^; 0.35 ± 0.086 mm^2^, respectively; *p* = 0.0025) (Figure 3).

The left and right transverse sinuses (LTS, RTS) were also enlarged, with the mTBI and sham values being 0.17 ± 0.045 vs. 0.089 ± 0.012 mm^2^, respectively (*p* = 0.0013) for LTS and 0.17 ± 0.046 vs. 0.091 ±0.012 mm^2^, respectively (*p* = 0.0019) for RTS (Figure 3).

Thus, all three sinuses were significantly larger in the mTBI group compared to the sham. There were no significant differences between males (*n* = 5) and females (*n* = 7) from the mTBI group in the SSS volume (*p* = 0.61), SSS area (*p* = 0.90), LTS area (*p* = 0.25), or RTS area (*p* = 0.35).

### mTBI Rats From Study 2 Have Enlarged Sinuses One Day Post-injury

The enlarged SSS is seen on gad enhanced MRIs of 1 day mTBI rats (Figure 2).

**Figure 4:**
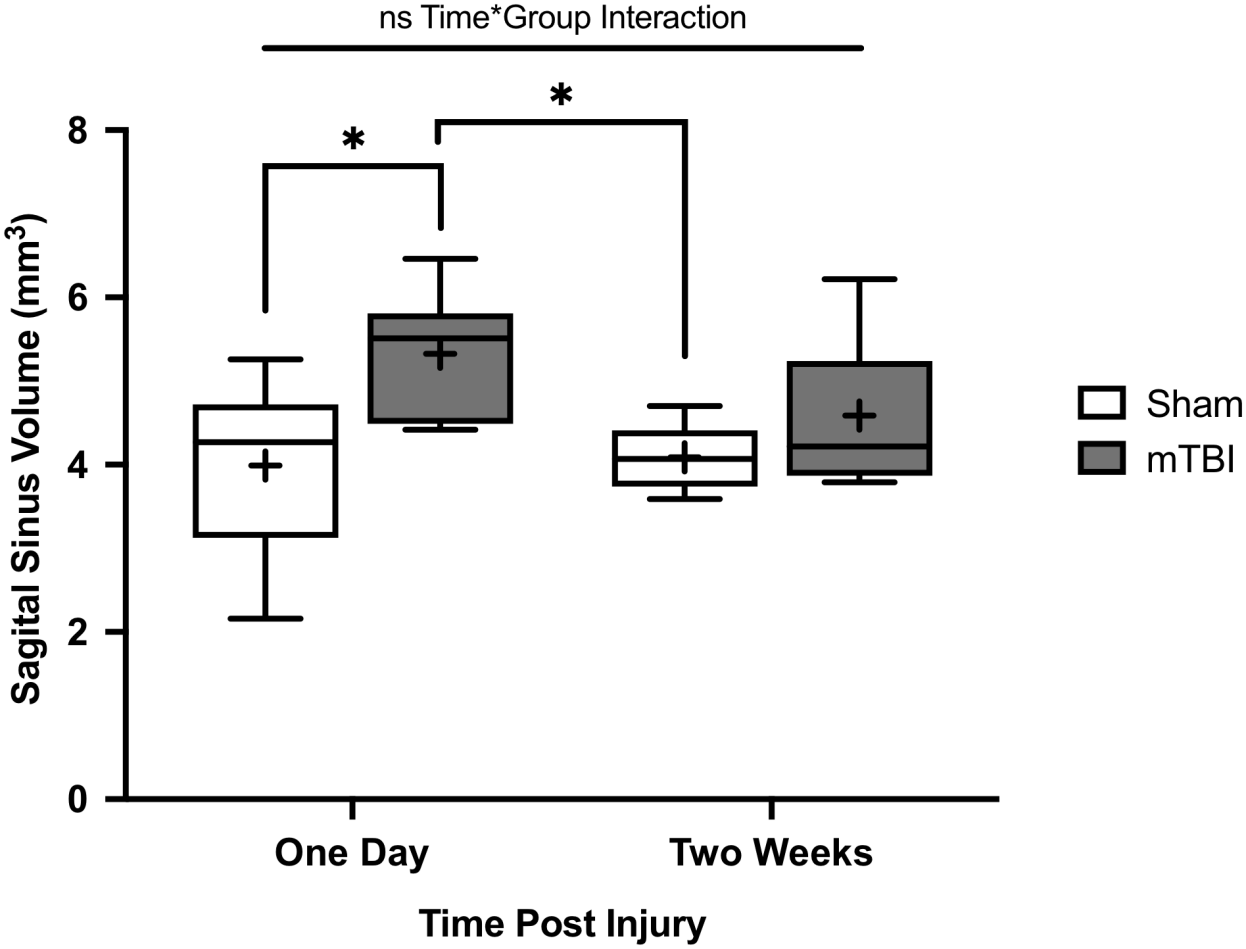
Superior sagittal sinus (SSS) volumes of rats from Study 2. Data were obtained from T1w Gadolinium enhanced MRI. Volumes were calculated from 10 mm of consecutive slices. Comparisons were done with a two-way ANOVA followed by Tukey *post-hoc*. Rats were imaged either 1 day post injury (mean±SD; Sham 1D *n* = 6; mTBI 1D *n* = 7) or 2 weeks post injury (mean±SD; Sham 2W *n* = 7; mTBI 2W *n* = 7). No significant interaction was found between the mTBI/sham group and time point (*p* = 0.19, *F* = 1.8). There was a significant difference between mTBI and sham rats (*p* = 0.005, *F* = 9.8) and no significant differences between 1 day and 2 week rats (*p* = 0.31, *F* = 1.1). SSS volume was significantly increased for 1-day mTBI rats compared to the 1-day (*p* = 0.030) and 2-week (*p* = 0.038) sham rats. No significant differences for 2-week mTBI rats compared to 2-week sham rats (*p* = 0.66) or 1-day mTBI rats (*p* = 0.33). *p < 0.05.

Overall, SSS volume was 4.0 ± 1.1 and 5.3 ± 0.78 mm^3^ for 1 day sham and mTBI rats respectively. Two-week sham and mTBI rats had SSS volumes of 4.1 ± 0.38 and 4.6 ± 0.90 mm^3^, respectively. A two-way ANOVA showed no significant interaction between mTBI/sham group and time point (*p* = 0.19, *F* = 1.8). SSS volume was found to be significantly increased in mTBI rats compared to shams (*p* = 0.005, *F* = 9.8), while no significant difference was found between 1 day and 2 week rats (*p* = 0.31, *F* = 1.1) (Figure 4). Tukey *post-hoc* comparisons between groups found a significant increase in volume for 1 day mTBI rats compared to 1 day sham rats (*p* = 0.030), and 2 week sham rats (*p* = 0.038) (Figure 4). We found no significant difference for 2 week mTBI rats compared to 2 week sham rats (*p* = 0.66), or 1 day mTBI rats (*p* = 0.33) (Figure 4).

## Discussion

Closed head impact models are currently a common model for studies of mTBI or concussion (20). In particular, a modification where the head is not fixed and can rotate freely is thought to create an injury that is more similar to what can occur during rotational/translational impacts believed to generate mTBI (17, 21). A key component of these models, in terms of relating to mTBI in humans, is that they exhibit no significant focal injury in the brain (22). We confirmed this with MRI, in that the cortex of the brain lacked any visible hemorrhage or edema in our rats (Figure 2).

There are two reasons for there being two studies (models) in this paper. One of the most common models is the vertical drop closed head impact model (17). In this model, the impactor will strike the middle of the sagittal sinus (23). This leads to the possibility that the vertical weight drop could cause direct damage to sagittal sinus. In order to control for this possibility, we also used a lateral impact model (Study 2), where the impactor did not strike near the superior sagittal sinus. The lateral impact model incorporated multiple hits as repeated injury, which is also becoming a common study design (24).

The second reason for undertaking Study 2 relates to the MRI method itself. In Study 1, we used T2w MRI and drew an ROI around the darkening of the SSS. Darkening in venous drainage vessels is caused by increased magnetic susceptibility introduced by deoxyhemoglobin in the vasculature. This susceptibility, also known as a blooming effect, can extend beyond the area of the lumen of the vessel (25). As blooming can intensify with increased deoxyhemoglobin content, it was possible that a significantly increased area could be caused by decreased oxygen saturation in the venous system. To control for this, we added Study 2, in which we used a completely different MRI method to assess lumen area. We used gadolinium enhanced MRI and a T1w image. This method is much less sensitive to the effects of deoxyhemoglobin.

The fact that we saw the dilation in both Study 1 and Study 2, where the impactor contacted vertically or laterally, indicates that the change is not caused by direct impact on the sinus.

While overarching literature indicates that veins function through passive regulation, some suggest this might not be so in the cerebral venous system. Smooth muscle cells have been detected at the end of each cortical vein in human cadavers (26). These cortical veins possessed what seemed to be a myoendothelial sphincter, which was suggested to be involved in cerebral venous regulation (26). Smooth muscle in cortical veins is evidence for venous regulation. The retroglenoid vein in dog brain has also been reported to have sphincter-like attributes and innervation (27). Nerve stimulation resulted in diversion of venous flow to alternative pathways (27). Both studies present compelling evidence for venous regulation in large draining vessels of the brain.

We detected dilation of venous sinuses post-mTBI. Increased CBF could potentially result in a transient increase in diameter of venous sinuses. However, an arterial spin labeling study, done for an MSc thesis, demonstrated no differences in perfusion in the model used in Study 1 (28). This observation warrants repeating. The next most likely cause for dilated sinuses would be a constriction at some point downstream in the venous system.

A study on patients with mTBI supports the suggestion that there is abnormal venous drainage after injury. Constriction of the internal jugular vein (IJV) was observed after mTBI (16). This was compensated for with increased venous outflow through the secondary veins (16). mTBI patients also experienced a decrease in intracranial compliance and an increase in intracranial pressure (16). The data were obtained using MR venography in adult subjects ranging from 6 months to 29 years post injury, indicating these changes may be sustained long term. Constriction of the IJV could impair venous drainage. We propose that this constriction may prevent venous blood from draining properly, thereby resulting in dilated sinuses.

In our study, the sinus enlargement was observed at the 24-h timepoint but not the 2 week timepoint. As sinus enlargement may be indicative of altered venous drainage, our findings are in accordance with the above described human study. However, it differed from the above study in that sinus dilation in our rats resolved sometime after the 24 h timepoint. It is possible that the type of injury or magnitude of the injury may relate to the duration of abnormal flow regulation. This would need further research.

Distention of the major cerebral venous sinuses is evidence of altered venous regulation, which may be a potential physiological response to mTBI. It is easy to imagine how distended vessels could relate to meningeal pain and headache. There is a larger implication in that these data suggest that venous flow regulation may be possible in mammals. Further research is warranted to understand the prevalence of enlarged sinuses, as well as the mechanisms causing the dilation.

## Data Availability Statement

The datasets generated for this study are available on request to the corresponding author.

## Ethics Statement

The animal study was reviewed and approved by The Animal Care Committee (University of Calgary) for Study 1 and the University of Calgary Conjoint Faculties Research Ethics Board for Study 2.

## Author Contributions

QS and AH wrote the manuscript with oversight from JD. QS and AH conducted data analysis and interpretation of both studies. EI was involved in Study 1 design, data collection, and interpretation. JC was involved in Study 2 design and data collection. RM developed the study design for Study 1 and 2 and was involved in data collection for both. JD reviewed the study design, data analysis and interpretation for both studies. All authors contributed to manuscript revision, read, and approved the submitted version.

## Conflict of Interest

The authors declare that the research was conducted in the absence of any commercial or financial relationships that could be construed as a potential conflict of interest.
